# Combined lateral and ventral approach for 360° decompression of an intraosseous suprasellar meningioma encasing the optic nerve

**DOI:** 10.3171/2025.10.FOCVID25187

**Published:** 2026-01-01

**Authors:** Adway Gopakumar, Shovan Bhatia, Eric W. Wang, Georgios A. Zenonos

**Affiliations:** Departments of 1 Neurological Surgery and; 2 Otolaryngology, University of Pittsburgh Medical Center, Pittsburgh, Pennsylvania

**Keywords:** meningioma, optic nerve, endoscopic endonasal approach, pterional approach

## Abstract

Suprasellar meningiomas present operative challenges because of their involvement with cranial nerves and neurovascular structures. The optimal approach for suprasellar meningiomas encasing the optic nerve remains debated. This video presents a patient with profound vision loss limited to hand motion, arising from a calcified suprasellar meningioma with 360° optic nerve encasement, which was resected via an anterolateral approach with extradural anterior clinoidectomy followed by an endoscopic endonasal approach. Postoperatively, vision improved to 20/30 at 2 months with no recurrence at 2 years. This case highlights the complementary roles of transcranial and endonasal strategies in safely resecting an intraosseous suprasellar meningioma encasing the optic nerve.

The video can be found here: https://stream.cadmore.media/r10.3171/2025.10.FOCVID25187

## Transcript

This is a case of a combined lateral and ventral approach for a circumferential decompression of an intraosseous suprasellar meningioma encasing the optic nerve.

### 0:29 Clinical Presentation.

The case refers to a 50-year-old female who presented with gradual vision loss over the prior year. She had a family history of Stargardt’s disease as well as a personal history of atypical hyperplasia multiple years prior.

### 0:46 Physical and Neuro-Ophthalmological Exam.

 On visual examination, she had hand motions only on the left side with an afferent pupillary defect but normal vision on the right. Here are her visual fields and her optical coherence tomography showing significant thinning of the ganglion cell layer on the left side.

### 1:04 Neuroimaging Findings.

Her imaging showed an expansile mass centered on the planum and tuberculum filling the sphenoid sinus. This was mainly bony, but it had some soft components and was circumferentially encircling the optic nerve. You see that this was extending over the left anterior clinoid process and compressing the nerve both from medially and laterally. The lesion was significantly calcified, expansile to the native bone.

### 1:42 Other Findings.

She had normal pituitary panel and negative metastatic workup.

### 1:46 Approach Considerations.

We consider a lateral approach with anterior clinoidectomy, an endoscopic endonasal approach, a transbasal approach with a pericranial flap, as well as combined approaches with endonasal and lateral, exchanging which is done first.^1^

### 2:01 Approach Considerations.

An endoscopic approach here would provide direct visualization of the inferior medial optic canal, allowing for less nerve manipulation and also preservation of the correct arachnoidal planes, as it would be approaching the tumor from its origin. It also provides an excellent reconstruction option with nasoseptal flap for the large bony defect that would be resulting after resection of this tumor. However, optic nerve decompression would happen later in the case, potentially after blood loss, which would compromise the perfusion of the optic nerve, which is compressed and also without any significant landmarks, as most of the tumor is filling the sphenoid. It would also provide poor access to the anterior clinoid that provided the lateral compression in this case.^2^

### 2:49 Approach Considerations.

On the flip side, a pterional craniotomy with an anterior clinoidectomy would provide early bone decompression of the optic nerve with well preserved landmarks and before any significant blood loss has occurred, which would compromise the perfusion of the optic nerve. It would also help us remove the lateral compression but provides poor and blind access to the inferomedial optic canal as well as attacks the tumor from the wrong arachnoidal planes, potentially placing the perforators to the optic nerve at risk from the superior hypophyseal arteries.^3^

### 3:29 Combined Approach.

We chose to perform a combined approach starting with the lateral first, as this would provide early decompression of the optic nerve with preservation of landmarks and before any significant blood loss, and perform secondly an endonasal approach, which would allow us to approach the inferomedial optic canal, which is commonly affected in these cases, without significant manipulation of the optic nerve. It would allow us to preserve the correct arachnoidal planes and also would give us a very good reconstructive option.

### 4:05 Sequential Approach Rationale.

We chose to perform this procedure in a sequential, as opposed to a staged or simultaneous, fashion as a compromise between being expeditious and avoiding two trips to the OR, but also having control of blood loss and having our full attention at each task at hand.

### 4:27 Neuroanesthesia Considerations.

Good neuroanesthesia considerations for cases like this are a preinduction A-line with maintenance of mean arterial pressures above 80; maintenance of euvolemia and avoiding hypovolemia; placing a lumbar drain up front allows brain relaxation for the extradural anterior clinoidectomy without requiring high doses of mannitol, which may cause hypovolemia and compromise the profusion of the optic nerve; steroids for optic nerve protection, as well as being very mindful of the hematocrit and the blood loss during the case.

### 5:01 Patient Positioning.

The positioning was one that would accommodate both a lateral as well as an endonasal approach done in sequence.

### 5:10 Preoperative Imaging.

Again, the tumor here was circumferentially compressing the optic nerve with extension in the left anterior clinoid process. We were starting with a lateral approach first, with a relatively limited frontolateral approach for an anterior clinoidectomy. Placement of a lumbar drain preoperatively with drainage above approximately 80 cc of cerebrospinal fluid.

### 5:39 Lateral Approach.

Here’s the approach of drilling the anterior clinoid and skeletonizing the optic nerve. I’m drilling a trough medial to the optic nerve, which would serve as a landmark when we come laterally. Here we see the periorbita, the optic nerve, the supraorbital fissure, optic strut, and carotid-oculomotor membrane that were exposed. Again, drilling a trough medially to the optic nerve will provide a good landmark so that we don’t land on the optic nerve when we’re coming ventrally without good landmarks.

### 6:13 Ventral Approach.

Coming endonasally, a nasoseptal flap was elevated, there’s soft components to it, but also a significant amount of hyperostotic bone. We’re finding here the medial landmark, the medial trough that is a landmark in exposing the sella; it’s our goal to find and extrapolate anatomy. Eggshelling the bone and removing it slowly. Here we are drilling out the lamina papyracea that was significantly hyperostosed and extending back to the lateral opticocarotid recess with the extension of the optic strut from the other side, from the endonasal side.

### 6:49 Optic Nerve Decompression.

Here, I’m removing it completely, essentially achieving a 360° bony decompression of the optic canal, extending the bony decompression on the right side as well, and finally opening the dura.

Our goal here is to again maintain the microvasculature of the optic nerve. Here’s the invasion of the inferomedial optic canal, and direct visualization of that interface between the optic and the tumor is crucial for maintaining those small perforators to the optic that can be quite detrimental if hurt. Extending the exposure to the distal dural ring and trying to trim the dura of the distal dural ring back to the ophthalmic artery.

### 7:36 Tumor Resection.

Here we’re removing some of the tumor that’s invading the superior intracavernous sinus and cutting further down to remove the diaphragma sellae that was involved with soft tumor. Taking back the diaphragmatic cut to the stalk and further trimming again of the distal dural ring.

### 7:59 Tumor Resection.

Here we’re opening into the cavernous sinus after Dopplering the carotid artery to facilitate further resection of the diaphragma and some of the tumor that was extending into the cavernous sinus. Finally, trimming some of the tumor extending to the prechiasmatic recess and planum and coagulation of the dural attachment.

### 8:19 Final Result.

Here’s the final result of a good resection of the tumor, circumferential decompression of the optic nerve with maintenance of the microvasculature of the optic nerve.

### 8:29 Closure.

For reconstruction, we perform a multilayered reconstruction with an inlaid dural substitute, a fascial allograft, and a vascularized nasoseptal flap, which as you see here has good perfusion on indocyanine green angiography, absorbable and nonabsorbable packing.

### 8:49 Postoperative Course.

Here’s the postoperative imaging showing bony 360° decompression of the optic nerve. The patient had an uneventful course, had a lumbar drain for 72 hours, and was discharged on postoperative day 3 without any complications and with immediate subjective improvement in vision.

### 9:07 Postoperative Findings.

At 2 months postoperatively, she had improved to 20/30 and was able to read from hand motions, that was her preoperative exam. Here are her visual fields postoperatively, and at 2 years postop, there was no evidence of recurrence of the tumor.^4,5^

### 9:27 Conclusion.

In conclusion, optic nerve compression should be approached with caution before, during, and after surgery, avoiding excessive manipulation and decreased perfusion of the nerve, and taking every precaution to avoid injury to the microvasculature.^6^ Combined approaches can sometimes provide optimal decompression when the nerve is compressed circumferentially, but appropriate case selection and attention to detail remains crucial.

## Disclosures

The authors report no conflict of interest concerning the materials or methods used in this study or the findings specified in this publication.

## Author Contributions

Primary surgeon: Zenonos. Assistant surgeon: Wang. Editing and drafting the video and abstract: Zenonos, Gopakumar, Bhatia. Critically revising the work: Zenonos, Gopakumar, Bhatia. Reviewed submitted version of the work: all authors. Approved the final version of the work on behalf of all authors: Zenonos. Supervision: Zenonos.

## Supplemental Information

### Patient Informed Consent

The necessary patient informed consent was obtained in this study.
